# Post-Operative Physical Therapy Following Cervical Spine Surgery: Analysis of Patient-Reported Outcomes

**DOI:** 10.7759/cureus.40559

**Published:** 2023-06-17

**Authors:** Nathan A Lorentz, Matthew S Galetta, Michelle A Zabat, Tina Raman, Themistocles S Protopsaltis, Charla Fischer

**Affiliations:** 1 Orthopaedic Surgery, New York University (NYU) Grossman School of Medicine, New York, USA

**Keywords:** patient reported outcomes, promis scores, physical therapy, cervical disc replacement, anterior cervical discectomy fusion

## Abstract

Introduction

Post-operative physical therapy (PT) following anterior cervical discectomy and fusion (ACDF) surgery is often performed to improve a patient's functional ability and reduce neck pain. However, current literature evaluating the benefits of post-operative PT using patient-reported outcomes (PROs) is limited and remains inconclusive. Here we compare post-operative improvement between patients who did and did not undergo formal PT after ACDF using Patient-Reported Outcomes Measurement Information System (PROMIS) scores.

Methods

A retrospective observational study examining patients who underwent one- or two-level primary ACDF or cervical disc replacement (CDR) at an academic orthopedic hospital and who had PROMIS scores recorded pre-operatively and through two-year follow-up. Patients were stratified according to whether or not they attended formal postoperative PT. PROMIS scores and patient demographics were compared using the Mann-Whitney U test, Fisher’s exact test, chi-square test of independence, and Student’s t-test within and between cohorts.

Results

Two hundred and twenty patients were identified. Demographic differences between PT and no PT groups include age (PT 54.1 vs. no PT 49.5, p=0.005) and BMI (PT 28.1 vs. no PT 29.8, p=0.028). The only significant difference in post-operative PROMIS scores was in physical health scores at three months post-operatively (no PT 43.9 vs. PT 39.1, p=0.008). Physical health scores improved from baseline to one-year follow-up in both cohorts (PT +3.5, p=0.025; no PT +6.6, p=0.008). There were no significant differences when comparing improvements in physical health scores between groups at six months and one year.

Conclusion

In conclusion, there was no significance to support the benefits of post-operative PT as measured by PROMIS scores. No significant differences in PROMIS were observed between groups from pre-operative baseline scores to six-month and one-year follow-ups.

## Introduction

Surgical spine procedures are commonly used to treat spinal injuries and degenerative diseases that fail to respond to conservative management. Some of the most prevalent spinal degenerative disorders occur in the cervical spine and cause significant pain and dysfunction in patients [1]. Innovations in surgical technique now allow for successful relief of conditions, including cervical radiculopathy and myelopathy, through procedures, including anterior cervical discectomy and fusion (ACDF) or cervical disk replacement (CDR) [2-4]. The pain relief, functional improvement, and patient satisfaction these procedures provide have been well-proven through both retrospective and prospective studies [3,5-13]. For example, Andresen et al. found that a majority of patients reported significant positive changes in their health status (74.3%, as measured by the SF-36), had significant improvements in their pain levels, and reported high rates of satisfaction [12].

Post-operative physical therapy (PT) is an integral aspect of care following most orthopedic surgeries, including total hip and knee arthroplasties. There is, however, no consensus regarding the optimal timing and degree of PT in patients who undergo ACDF or CDR. Patients undergoing spine surgery often have considerable difficulty returning to normal mobility levels. This is thought to be due to many factors, such as pain, fear, deconditioning due to extended periods of pain-limited activity, and associated motor control deficits due to neurologic damage [12,14-16]. As such, physical therapy is not necessarily included in the rehabilitation protocol for patients who have undergone spine surgery. As the number of cervical spine surgeries performed annually in the U.S. continues to increase, evidence-based practices aimed at improving patient outcomes are becoming increasingly important.

The current literature evaluating the benefits of post-operative PT using patient-reported outcomes (PROs) is limited and remains inconclusive [17-19]. Additionally, PROs have historically been measured using such tools as general health questionnaires (Medical Outcomes Study Short Form), pain rating scales (VAS), or disease-specific questionnaires (NDI, Oswestry Disability Index). These measurement tools have limitations that include decreased precision, interpretability, and inter-survey comparability. To overcome these limitations, the PROMIS (Patient-Reported Outcomes Measurement Information System) system was developed and allows for the measurement of outcomes across more granular categories such as mental health, physical health, and pain interference and intensity [20-22].

The majority of studies evaluating ACDF and CDR have focused primarily on the surgical technique itself or peri-operative outcomes and complications rather than the impact of post-operative interventions to improve overall outcomes. Additionally, no prior study has investigated the benefits of post-operative PT following ACDF and CDR using PROMIS scores as the measurement metric. The purpose of this study is to compare post-operative improvement between patients who did and did not undergo formal PT after ACDF or CDR using PROMIS scores.

## Materials and methods

Study design

This study was a retrospective review of consecutive patients presenting to a single urban academic orthopedic hospital from 2013 through 2020. It was approved by the New York University Grossman School of Medicine Institutional Review Board (IRB) under study number 18-00668. Inclusion criteria were patients 18 years or older who underwent primary, elective, one- or two-level ACDF or CDR. Patients also required PROMIS scores recorded pre-operatively as well as at postoperative time points of interest (two weeks, six weeks, three months, six months, one year, two years) to be included. Exclusion criteria included patients with three or more levels fused. No funding was received for this study, and thus funding played no role in the design, conduct, or reporting of this study.

PROMIS computer adaptive test

The PROMIS computer-adaptive test measures individual outcomes of interest. It is a standardized patient-reported outcome measurement system that is standardized to account for differences in age and sex among respondents [22]. In this study, PROMIS metrics of interest were mental health, physical health, pain interference, pain intensity, mobility, physical function, and upper extremity function. For each PROMIS metric, the mean is scored at 50, and each 10-point deviation reflects one standard deviation change of improving or worsening function.

Data collection

Patient demographics of age, sex, BMI, smoking status, and race were included and compared. PROMIS scores for the aforementioned domains were obtained pre-operatively and at two-week, six-week, three-month, six-month, one-year, and/or two-year follow-ups. Patients were divided into two cohorts for analysis based on whether or not they attended formal PT sessions.

Statistical analysis

IBM Corp. Released 2017. IBM SPSS Statistics for Windows, Version 25.0. Armonk, NY: IBM Corp. was used to analyze the collected data. The Mann-Whitney U test was conducted for continuous data (PROMIS scores). Fisher’s exact test was conducted for categorical data (gender, smoking, and race). A chi-square test of independence was conducted for discrete data (procedure type, levels instrumented). A Student’s t-test was used to compare the improvement in postoperative PROMIS scores from baseline between PT and no PT patients.

## Results

Patient demographics

In total, 220 patients were identified. Demographic differences between PT and no PT groups include age (PT 54.1 years vs. no PT 49.5 years, p=0.005) and BMI (PT 28.1 kg/m2 vs. no PT 29.8 kg/m2, p=0.028). There was no baseline relationship to the incidence of PT based on either procedure type or the number of levels instrumented (Table 1).

**Table 1:** Demographic Information Bolded p-values indicate statistical significance. ACDF: Anterior Cervical Discectomy and Fusion. CDR: Cervical Disc Replacement. BMI: Body Mass Index. PT: Physical Therapy

Demographic	PT	No PT	p-value
Number of Patients	139	81	N/A
ACDF	109	56	0.13
CDR	30	25	
1 Level Procedures	88	48	0.55
2 Level Procedures	51	33	
Age	54.1	49.5	<0.01
Sex (Male %)	43 (53.1%)	82 (59.0%)	0.39
Current Smoker	12	25	0.38
BMI	28.1	29.8	0.03
Race (White, African American, Asian, Other)	94/17/9/19	58/9/5/9	0.93

PROMIS scores

When comparing mean PROMIS scores between the PT and no PT cohorts at two-week, six-week, three-month, six-month, one-year, and two-year postoperative follow-up, the only significant difference seen was in the physical health score at three-month postoperative follow-up, with the no PT group having a significantly higher score than the PT group (no PT 43.9 vs. PT 39.1, p=0.008). Otherwise, all other scores at the various follow-up intervals were not significantly different between groups (Table 2).

**Table 2:** Postoperative PROMIS Scores Between Groups Bolded p-values indicate statistical significance. PT: Physical Therapy. UE: Upper Extremity. PROMIS: Patient-Reported Outcomes Measurement Information System.

Pain Intensity	PT	No PT	p-value
6 months - mean (# of patients)	47.9 (27)	49.5 (21)	0.58
1 year - mean (# of patients)	48.3 (45)	51.1 (28)	0.19
Pain Interference	PT	No PT	p-value
6 months - mean (# of patients)	57.8 (30)	59.4 (22)	0.57
1 year - mean (# of patients)	59.4 (48)	62.0 (28)	0.21
UE Function	PT	No PT	p-value
6 months - mean (# of patients)	33.5 (10)	38.4 (10)	0.09
1 year - mean (# of patients)	38.4 (18)	35.4 (11)	0.44
Physical Function	PT	No PT	p-value
6 months - mean (# of patients)	45.3 (15)	41.8 (10)	0.37
1 year - mean (# of patients)	40.4 (25)	37.3 (15)	0.44
Mobility	PT	No PT	p-value
6 months - mean (# of patients)	41.5 (10)	42.4 (9)	0.84
1 year - mean (# of patients)	43.8 (14)	41.7 (7)	0.5
Mental Health	PT	No PT	p-value
Preoperative - mean (# of patients)	44.6 (42)	43.5 (19)	0.71
2 weeks - mean (# of patients)	45.2 (35)	49.1 (24)	0.13
6 weeks - mean (# of patients)	46.7 (41)	49.3 (34)	0.24
3 months - mean (# of patients)	43.9 (48)	48.3 (33)	0.3
6 months - mean (# of patients)	46.1 (64)	46.5 (51)	0.83
1 year - mean (# of patients)	46.9 (97)	46.6 (52)	0.86
2 years - mean (# of patients)	45.4 (56)	43.0 (31)	0.31
Physical Health	PT	No PT	p-value
Preoperative - mean (# of patients)	38.2 (42)	35.9 (19)	0.19
2 weeks - mean (# of patients)	39.7 (35)	41.1 (24)	0.46
6 weeks - mean (# of patients)	40.5 (41)	42.8 (34)	0.16
3 months - mean (# of patients)	39.1 (48)	43.9 (33)	<0.01
6 months - mean (# of patients)	40.8 (64)	42.4 (50)	0.31
1 year - mean (# of patients)	41.7 (96)	42.5 (51)	0.61
2 years - mean (# of patients)	40.0 (55)	38.5 (30)	0.46

When comparing mean PROMIS scores within the PT and no PT cohorts at the same follow-up time points, sufficient pre-operative baseline PROMIS scores were only available for the domains of physical health and mental health. In these domains, physical health scores were seen to improve significantly over baseline by six-month follow-up in the no-PT cohort (p=0.006); improvement for the PT cohort at this time was not significantly improved compared to baseline (p=0.13). However, by the one-year follow-up mark, physical health scores were seen to improve from the pre-operative baseline in both the PT and no PT groups (PT +3.5, p=0.025; no PT +6.6, p=0.008). There were no significant improvements from baseline in the domain of mental health at any follow-up time point (Table 3). 

**Table 3:** Improvements in Postoperative PROMIS Scores From Baseline Within and Between Groups Bolded p-values indicate statistical significance. PT: Physical Therapy. PROMIS: Patient-Reported Outcomes Measurement Information System.

	Improvement in Mental Health Score				Differences in Improvement
	PT	p-value	No PT	p-value	p-value
Preoperative - 6 Months	1.5	0.48	3.0	0.24	0.84
Preoperative - 1 Year	2.3	0.29	3.1	0.22	0.73
	Improvement in Physical Health Score				Differences in Improvement
	PT	p-value	No PT	p-value	p-value
Preoperative - 6 Months	2.6	0.13	6.5	<0.01	0.23
Preoperative - 1 Year	3.5	0.03	6.6	<0.01	0.36

## Discussion

PT is a widely utilized post-operative rehabilitation intervention for patients undergoing ACDF or CDR. However, little is known about the effect of post-operative PT on outcomes following these procedures. To our knowledge, there have been no published studies assessing the impact of PT on patient pain and functional recovery following surgery using PROMIS scores. While further research is necessary to more fully characterize the impact of physical therapy on postoperative recovery as well as the populations in which postoperative PT provides benefit, the results of this study suggest PT may not be a critical universal recommendation across all patients undergoing ACDF or CDR.

In this retrospective analysis of 220 patients, we assessed cohorts who did and did not receive PT according to PROMIS scores collected pre-operatively and during post-operative follow-up. Our findings suggest that at three months post-op, patients who had not received PT had significantly greater improvements in physical function compared to patients who had received PT, although improvements in both cohorts at this time were not significant compared to baseline. This discrepancy was resolved by the six-month time point, and by one year, both cohorts had made improvements in physical function from baseline that were statistically significant within each cohort but similar between cohorts. In the other domains of interest (pain intensity, pain interference, upper extremity function, mobility, mental health, and physical health), there were no significant differences between cohorts at any time point.

The results of our study were similar to those found in the studies conducted by Peolsson et al. and Wibault et al. [18,19]. In our study, both groups experienced similar significant improvements in their physical health PROMIS scores from pre-operative baseline to one-year follow-up. Similarly, when comparing structured PT and a standard post-operative approach by six-month follow-up, Wibault et al. found that there were no significant differences in neck disability index (NDI) and visual analog scale (VAS) pain scores between groups but that both groups did experience improvements from their pre-operative baseline scores [18]. Peolsson et al. similarly found no significant between-group effects for the NDI or VAS scores at two-year follow-up. There were also no significant differences between secondary outcome measures of neck/arm pain intensity and frequency and health-related quality-of-life variables [19]. In contrast to our results, however, all outcome measures showed a significant improvement over time in both groups. Comparison between the present study and these prior studies is limited by the fact that, in the Wibault et al. and Peolsson et al. studies, rather than comparing between a formal PT group and no PT group, their comparison group received a “standard post-operative approach” in which PT was accessed as needed, and patients sought PT without needing a referral. 61% of patients in the standard post-operative approach cohort reported additional use of PT, which significantly weakens the quality of their evidence. In our study, we included only patients who did not access PT at all in our no-PT cohort.

Despite the widespread recommendation for PT after orthopedic surgical procedures, there is a mixed body of evidence on the benefit of postoperative PT or the mechanisms underlying any advantages. In the case of PT after ACDF or CDR, the limited gains in physical function may reflect a suboptimal set of PT exercises for this particular pathology or intervention rather than an intrinsic lack of benefit from PT overall. Additionally, though demographics between cohorts were similar aside from age and BMI, there may be some other intrinsic patient factor influencing participation in PT that was not captured in the PROMIS metrics of interest. Perhaps most actionable is the observation that there were no differences between the cohorts in physical function scores at two weeks and six weeks, time points that both fall into the post-operative period when decisions about physical therapy are being made. This may suggest that the decision to undergo PT or not should be made with strong consideration of the patient’s perception of their own goals, recovery, and resources in order to best meet the patient’s expectations and achieve optimal satisfaction.

The present study is not without limitations. Though the PT cohort participated in a formal PT program, our data is unable to assess the degree of engagement by each participant within the PT sessions. Differences in ability or motivation to challenge oneself during sessions may obscure the significant benefits of PT in highly engaged patients. Additionally, larger sample sizes could provide a more robust understanding of outcomes trends for these patient populations. Although the standardization of PROMIS scores accounts for inter-respondent age and gender differences, cohort matching according to co-morbidities may help reduce selection bias. Finally, multi-level procedures were excluded from the study due to the heterogeneity of the procedures; further study to understand the role of PT in patients undergoing these interventions will help elucidate further trends.

## Conclusions

The computer-adapted PROMIS tool enables evidence-based comparison of post-operative rehabilitation after ACDF, which may inform the use of these interventions. PROMIS scores showed comparable improvements in physical function over baseline at one-year follow-up regardless of PT participation; there were no differences in other outcome domains. The variability in patient-reported perceptions of benefits from post-operative PT suggests that shared decision-making around whether an individual patient requires PT is the ideal approach to achieving patient expectations and optimizing patient satisfaction after surgery. Moreover, it suggests that the greatest benefit may be derived from surgeons and physical therapists working closely together to identify the particular post-operative rehabilitative strategy that best aligns with the patient’s pathology and the intervention they received.
